# Autistic People's Access to Bilingualism and Additional Language Learning: Identifying the Barriers and Facilitators for Equal Opportunities

**DOI:** 10.3389/fpsyg.2021.741182

**Published:** 2021-09-22

**Authors:** Rachael Davis, Sue Fletcher-Watson, Bérengère G. Digard

**Affiliations:** ^1^Salvesen Mindroom Research Centre, University of Edinburgh, Edinburgh, United Kingdom; ^2^Patrick Wild Centre, University of Edinburgh, Edinburgh, United Kingdom

**Keywords:** autism, bilingualism, wellbeing, language learning, inclusion

## Abstract

Bilingualism is a valuable tool that enriches and facilitates cultural, social and lived experiences for autistic and non-autistic people alike. Research consistently finds no negative effects of bilingualism and highlights the potential for positive effects across cognitive and socio-cultural domains for autistic and non-autistic children. Yet parents of autistic children remain concerned that bilingualism will cause delays in both cognitive and language development and are still frequently advised by practitioners to raise their child monolingually. Evidently, findings from research are not reflected in practice or subsequent advice, and it is essential to identify ways to ensure equal access to additional language learning. We briefly summarise the existing literature on bilingualism and autism, considering perspectives from the bilingual autistic community, and experimental research. We identify the most pertinent barriers to participation for autistic bilingual children in terms of familial, clinical and educational perspectives. We propose novel solutions to promote additional language learning and suggest changes to practice that will contribute to an evidence base for families and practitioners. This commentary makes innovative recommendations at both the individual and societal level to ensure that autistic bilingual people have equal rights and opportunities to language learning and are optimally supported in accessing them.

## Introduction

While all children should have access to language learning and the opportunities that come with it, as a fundamental human right, this is currently not the case for all autistic children. The U.N. Convention on the Rights of Persons with Disabilities (UN General Assembly, 2006)—which highlights rights for children—includes “respect for the right of children with disabilities to preserve their identities” and “recognising specific cultural and linguistic identity” (article 3). Although it could, in some instances, be reasonable to violate this right if there was evidence that bilingualism was harmful for development, no research to date has found long-term negative effects of bilingualism. Additionally, there is evidence to suggest that bilingualism can be beneficial to both autistic and non-autistic children. Indeed, a growing body of research highlights the potential benefits of bilingualism for non-autistic children in terms of sociocultural factors, including family bonds (Opitz and Degner, 2012) and wellbeing (Halle et al., 2014). When bilingual parents raise their children monolingually, there is also the possibility that children can be excluded from bonding with relatives and participating in cultural activities (Jegatheesan, 2011; Park, 2014). Findings are less consistent regarding cognitive benefits, in part because of the confounding factors in the field, such as the heterogeneity of demographic characteristics of bilingual people—i.e., socio-economic status and ethnic minority status—particularly in high-income countries.

While comparatively, there is limited research addressing the effects of bilingualism for autistic people, in terms of cognitive skills, the literature that does exist suggests bilingual exposure does not lead to poorer development. Research assessing executive functions, social cognition and language abilities of autistic people suggest no detrimental effects of bilingualism (e.g., Beauchamp and MacLeod, 2017), and some preliminary indications of positive effects (e.g., Valicenti-McDermott et al., 2013; Gonzalez-Barrero and Nadig, 2019; Montgomery et al., 2021), though more research is needed to quantify these claims. A detailed overview of the cognitive literature is outside the scope of this commentary, but see Drysdale et al. (2015) and Wang et al. (2018) for systematic reviews of this research.

Autistic children in particular could benefit from the close familial and cultural connections that their shared language affords (Yu, 2013; Hampton et al., 2017). As autistic children experience greater levels of adversity in terms of wellbeing and social exclusion (Humphrey and Symes, 2010), preventing another source of positive self-regard—access to the home language—could exacerbate this further. This could be particularly relevant for autistic individuals from ethnic minority populations or lower economic and social backgrounds, who may particularly benefit from immersion in the home language.

Two studies to date have investigated the lived experiences of autistic bilingual people, both highlighting multiple benefits. Howard et al. (2019) reported that bilingual children living in multilingual environments are more positive about bilingualism than their peers in monolingual settings, especially regarding social and communication opportunities linked with bilingualism. Adults also reported that bilingualism had supported their relationships with family and friends and increased their access to hobbies and educational and employment opportunities (Nolte et al., 2021). Importantly, bilingualism had given them feelings of increased self-efficacy, self-confidence and assurance in social interactions and had helped them to better understand themselves and others. Together, these findings highlight that bilingualism is not a burden, and can provide benefits across socio-cultural and cognitive domains for autistic people.

Despite an absence of negative effects of bilingualism (Uljarević et al., 2016), many parents remain concerned about potentially harmful effects of bilingualism on their autistic child's development. Parents are also frequently advised by clinical and educational practitioners to raise their child monolingually, sustaining the now-unsupported view that bilingualism could cause additional confusion or language delay (Kay-Raining Bird et al., 2012; Yu, 2013; Hampton et al., 2017). For example, one source for these concerns is the finding that bilingual children often have reduced vocabulary in each of their languages, even though their overall vocabulary across languages is comparable to that of their monolingual peers. Admittedly, a more restricted vocabulary in the majority language would impact the child's access to clinical support (Bialystok et al., 2010; Hoff and Ribot, 2017). However, research also shows exposure to multiple languages from early childhood is beneficial for language development, with some linguistic skills transferring across languages (e.g., Verhoeven, 2007).

Although research in this area is consistent in its findings cross-culturally and using a variety of different experimental paradigms, it is evident that research is not informing practise or filtering down to parents. In turn, this is leading to autistic children facing barriers to participation. To address this problem, we need to chart the barriers to additional language learning so that they can be overcome.

In this commentary, we identify the main barriers to inclusion in three areas—parental, clinical, and educational—and identify potential solutions to ensure equal access to additional language learning for autistic people.

## Familial Context

### Family Barriers

Specific concerns can arise in predominantly monolingual countries with parents who have a different native language (e.g., immigrant families) but choose to use the majority language at home. Unlike fluent speakers (Hudry et al., 2018), non-fluent speakers of the majority language report difficulty and discomfort communicating with their child and worry about teaching them a “wrong” way to speak the language, which can lead to reduced communication with their child (Kay-Raining Bird et al., 2016). One US-based study with immigrant parents of autistic children who only used English at home showed a decline in parent-child interactions and in the child's participation in family conversations (Kremer-Sadlik, 2005). Given the association between one's native language and emotional processing (Opitz and Degner, 2012), parents may feel more able to connect emotionally with their child in their native language, and choosing to speak a non-native language may undermine the emotional value of parent-child communication. Indeed, parents report feeling less restricted and closer to their autistic child when using their native language (Yu, 2013; Kim and Roberti, 2014). Choosing monolingualism can also have negative consequences on the child's feelings of identity, inclusion, and access to cultural heritage (Park, 2014).

This decision to stop using the home language is often motivated by the fact that the services a child might need would only be provided in the majority language (Kay-Raining Bird et al., 2012, 2016). As such, parents are often made to choose between access to services and the use of their native language with their child.

Additionally, findings suggest a lack of understanding of autism within some communities (Munroe et al., 2016; Hussein et al., 2019) which could also be compounded with social stigma reported by cultural minorities in the UK. In particular, experiences of increased stigma when accessing healthcare (Szczepura, 2005; Kandeh et al., 2020), and stigma around autism within their own and the wider community (Kinnear et al., 2016; Selman et al., 2018; Papoudi et al., 2021), could ultimately lead to increased social exclusion and misunderstanding.

Overall, research suggests parents can be influenced by misinformation on both bilingualism and autism, and this “double hit” could mean that accessing multiple languages at home may be particularly difficult for some autistic bilingual children.

### Family Solutions

Solutions to the issues above can be found in providing families with suitable information about autism and bilingualism. First, increasing parents' understanding of autism could be important in reducing stigmatising views. For example, research using short videos to teach non-autistic adults about autism had a significant impact on reducing stigma, increasing knowledge of autism, and increased positive beliefs (Jones et al., 2021). Providing parents with accessible resources combining knowledge of autism with a review of current findings regarding bilingualism could ensure that recommendations from research reach the people who will have a prominent role in the language experiences of their children.

Second, researchers must ensure that information about language exposure does not focus solely on the cognitive effects of bilingualism, but also on familial bonds, identity, community, and social inclusion. Within this, we cannot support an individual's cultural and linguistic identity without an understanding of their identity from their *own* perspective. It is crucial for children to feel involved when decisions are being made about and for them, and resources for families must also reflect the child's perspective. Currently, important questions remain around how autistic bilingual children feel about their language identity, feelings of inclusion with peers and within their cultural communities. Conducting research with children in this way will be an important step in creating a rigorous and complete evidence base for parents.

## Clinical Support

### Clinical Barriers

Research suggests that practitioners, including speech and language therapists, do not have confidence in the tools available to conduct diagnostic or language assessments with bilingual children (Davis et al., 2020). Although practitioners have a responsibility to maintain equality while conducting assessments, many standardised tools are culturally inappropriate for children from culturally and linguistically diverse populations (Mdlalo et al., 2019). The diagnostic process for bilingual children can bring additional challenges, with observational components of commonly used assessments being misinterpreted as autism-specific differences rather than differences in cultural norms. The absence of eye-contact and pointing behaviours, for example, are generally interpreted as autistic traits, yet in some non-western cultures, for a child to exhibit these behaviours with adults is inappropriate (Zhang et al., 2006). As a result, children from culturally and linguistically diverse populations are commonly diagnosed later (Shattuck et al., 2009) and are more likely to be misdiagnosed (Harris et al., 2014).

Similarly, some intervention strategies are not culturally relevant for all children. Practitioners noted that for some families, parent-child interactions are less direct or do not take place on the floor (Davis et al., 2020), children may be expected to display emotions differently, or the types of toys given to children to play with may not be typical in their culture (Norbury and Sparks, 2013). Recommending training or conducting assessments using these approaches may not be beneficial.

Practitioners themselves have stated they do not feel confident the tools available for use with culturally and linguistically diverse populations are acceptable, and frequently rely on their observations or asking for advice from colleagues or other professionals (Oxley and De Cat, 2019). Furthermore, speech and language therapists report that they have few opportunities to access additional cultural training after they qualify, and they do not have the time or opportunities to keep up to date with relevant research findings (Davis et al., 2020).

Importantly, the issues raised above regarding assessment difficulties for bilingual children mirror findings from research focusing on other neurodevelopmental conditions, such as developmental language disorders (Laasonen et al., 2018). Adapting bilingual-specific tools and practices would therefore benefit not only autistic children, but also children with other developmental conditions.

### Clinical Solutions

The solutions identified here relate to a larger tangible transformation that could optimise practice with autistic bilingual children and their families: providing co-produced resources, information, and training to ensure clinicians make confident and informed decisions about how to assess and support children in the diagnostic pathway and beyond. It is evident that more substantial and *ongoing* training opportunities should be available for practitioners.

As practitioners have raised concerns regarding the lack of cultural diversity training currently available, institutions should organise training as part of continual development. In the immediate future, researchers should ensure practitioners are provided with functional, up-to-date information about autism and bilingualism that can be integrated into training, to ensure parents are not choosing a monolingual environment because of practitioner uncertainty.

We must also consider ways of improving access to and understanding of the assessments currently offered. It is likely that where inappropriate recommendations and assessments are being used, practitioners have limited access to resources, such as developmental norms in specific languages or cultures (Oxley and De Cat, 2019). Therefore, providing practitioners with a wider range of assessment tools *and* information around the suitability of assessments and variability between children from culturally and linguistically diverse backgrounds would be the first step in preventing cultural biases. This can happen in three specific ways.

First, given the cultural variation in the identification and presentation of autism, research should focus on understanding and comparing the applicability and sensitivity of assessments across different cultures. To date, several autism screening tools have been developed for use with people from specific countries, including Iran (Samadi et al., 2014; Samadi and McConkey, 2015), China (Wang et al., 2020) and Brazil (Pacífico et al., 2019). All four studies highlighted a need to account for cultural variation, and that cultural and linguistic backgrounds play a crucial role in interpreting assessments.

Second, and of direct relevance to clinicians, is to create a checklist for common assessments to help practitioners analyse the appropriateness of such measures. For example, Harris et al. (2014) designed a checklist for four of the most common autism screening tools used with culturally and linguistically diverse children. By creating indicators of appropriateness that included compliance with disability requirements, it was evident some tools were more applicable than others. Future studies could develop comparable checklists that practitioners could access online and incorporate into assessments.

Third, the increased use of online diagnostic assessments that occurred during the COVID-19 pandemic could be leveraged to allow trained bilingual diagnostic practitioners to assess these children beyond their own health boards.

## Education

### Educational Barriers

In England, at least one in five pupils has English as an additional language, and this number is steadily rising (Leung, 2010; Department for Education, 2018). Despite this, a recent interview study with UK-based educators supporting autistic bilingual children (Howard et al., 2021) showed that although educators hold positive views about bilingualism, opinions vary greatly when it comes to autistic pupils. Many reported not recommending bilingualism for all autistic children, and this was predominantly based on the child's language abilities and autism profile. Educators believed that children with “*high functioning autism”* could choose the language they wanted to speak, but that for most autistic children bilingualism “*is not helpful,”* with potential confusion emerging as a major concern. Additionally, educators reported difficulty in effectively assessing and identifying the needs of autistic bilingual pupils. In special education, data regarding teachers' attitudes to bilingualism for autistic pupils is scarce, but no evidence suggests pupils with limited expressive language would not be able to understand words from different languages.

While some autistic bilinguals report learning their second language at school (Digard et al., 2020), autistic students can encounter barriers to modern language learning in mainstream education. There is a dearth of research addressing best practices for additional language learning for neurodivergent students, but evidence suggests that autistic pupils are often advised not to study modern foreign languages (Essex and MacAskill, 2020), which can penalise them by limiting their education, employment, and leisure opportunities (Nolte et al., 2021). This practice stems from a misunderstanding of autistic people's abilities, and a lack of understanding of autism. Without adequate training, educators find themselves unnecessarily adapting practises in an attempt to support autistic students, which could lead to unintentional exclusion.

### Educational Solutions

Monolingual educators, particularly those in monolingual environments, may underestimate the link between bilingualism, identity and inclusion, and a greater linguistic and cultural diversity amongst educators could help them better understand the experiences and needs of their autistic bilingual pupils (Yu and Hsia, 2019). Additionally, including dedicated training would help address some of the misconceptions expressed by educators (Iadarola et al., 2015; Howard et al., 2021) who often have limited experience with autistic bilingual pupils.

It is necessary to provide educators with information to make informed recommendations. The current strategy–focusing on the child's language abilities and autism profile–does not acknowledge the child's receptive language skills. Teachers who have supported autistic bilingual pupils over several years report that while children may experience early language difficulties, they often progress to fluency. Therefore, while a pupil's skills and profile should be considered, decisions should be made in a way that allows the child to develop as bilingual if they wish to do so. As assessing the needs and progress of these pupils can be challenging, developing suitable tools should become a priority area in education, which will also benefit non-autistic bilingual children.

Evidence regarding best practices to support autistic learners of modern languages in the classroom is lacking, and there is an undeniable need for more research in this area. However, there are anecdotal accounts of successful programs introducing foreign languages into classrooms with autistic students (Lumsden and Ruchill Autism Unit, 2009). However, there is a need for more specialised autism training for modern languages teachers, focusing on the best methods to prioritise autistic students' skills, while considering the areas of challenge (Wire, 2005).

## Conclusion

In this commentary, we identified the most pertinent barriers to participation for autistic bilingual children and young people. Many of these barriers have roots reaching further than the remits of clinical and educational settings, and stem from governmental policies failing autistic people. Public bodies lack awareness regarding the reality and diversity of the autistic population, and consequently, they fail to provide much-needed financial support for educators and clinicians. It is clear that additional resources are necessary to provide training opportunities that ensure optimal support for autistic bilingual people.

Impactful solutions in familial, clinical, and educational settings must be sponsored by local and national institutions through adapted policies. Providing appropriate educational and clinical support to autistic bilinguals requires practitioners to have access to updated and accessible information and training, and the financial support to develop research and support for educators and clinicians. Additionally, autistic bilinguals would greatly benefit from working with practitioners who can share their linguistic and neurodiverse experiences. Future policies should encourage and support bilingual and autistic bilingual students, particularly those from socially isolated communities who wish to enter educational or clinical careers, allowing them to utilise their own experiences. In summary, dedicated policies will be key to build a systemic change that will ensure autistic people can enjoy the same access to languages as their neurotypical peers.

Implementing such changes will be pivotal in ensuring access to language learning, which should be a fundamental human right for all children, and this will ensure that children who have the opportunity for dual language learning will benefit from equality of opportunity. This may include a richer and more inclusive cultural and social environment which in turn, could mean that children face fewer environmental barriers, allowing for greater individual autonomy and equal access to participation in society.

## Data Availability Statement

The original contributions presented in the study are included in the article/supplementary material, further inquiries can be directed to the corresponding author.

## Author Contributions

RD and BD: conceptualisation, writing–original draught, and writing–review and editing. SF-W: supervision and writing–review and editing. All authors made a substantial contribution to the writing of this paper.

## Funding

This work was supported by an Economic and Social Research Council Research Grant (ES/P00265X/1). The funding source had no role in the writing of the article or in the decision to submit the article for publication.

## Conflict of Interest

The authors declare that the research was conducted in the absence of any commercial or financial relationships that could be construed as a potential conflict of interest.

## Publisher's Note

All claims expressed in this article are solely those of the authors and do not necessarily represent those of their affiliated organizations, or those of the publisher, the editors and the reviewers. Any product that may be evaluated in this article, or claim that may be made by its manufacturer, is not guaranteed or endorsed by the publisher.

## References

[B1] Beauchamp M. L. MacLeod A. A. (2017). Bilingualism in children with autism spectrum disorder: Making evidence-based recommendations. Can. Psychol. 58:250. 10.1037/cap0000122

[B2] Bialystok E. Luk G. Peets K. F. Sujin Y. (2010). Receptive vocabulary differences in monolingual and bilingual children. Biling. Lang. Cogn. 13, 525–531. 10.1017/S1366728909990423 25750580 PMC4349351

[B3] Davis R. Sargent L. Zaki F. M. (2020). Autism and bilingualism: a thematic analysis of practitioners' experiences. OSF [Preprint]. 10.31219/osf.io/fcpk2 PMC13507578 37518984

[B4] Department for Education (2018). Schools, Pupils, and Their Characteristics: January 2018. Available online at: https://www.gov.uk/government/statistics/schools-pupils-and-their-characteristics-january-2018 (accessed July 27, 2021).

[B5] Digard B. G. Sorace A. Stanfield A. Fletcher-Watson S. (2020). Bilingualism in autism: language learning profiles and social experiences. Autism 24, 2166–2177. 10.1177/1362361320937845 32677446 PMC7549289

[B6] Drysdale H. van der Meer L. Kagohara D. (2015). Children with autism spectrum disorder from bilingual families: a systematic review. Rev. J. Autism Dev. Disord. 2, 26–38. 10.1007/s40489-014-0032-7

[B7] Essex J. MacAskill M. G. (2020). Modern foreign language education for learners with additional support needs in Scotland. Support Learn. 35, 440–453. 10.1111/1467-9604.12325

[B8] Gonzalez-Barrero A. M. Nadig A. S. (2019). Can bilingualism mitigate set-shifting difficulties in children with autism spectrum disorders? Child Dev. 90, 1043–1060. 10.1111/cdev.12979 29111575

[B9] Halle T. G. Whittaker J. V. Zepeda M. Rothenberg L. Anderson R. Daneri P.. (2014). The social–emotional development of dual language learners: looking back at existing research and moving forward with purpose. Early Child. Res. Q. 29, 734–749. 10.1016/j.ecresq.2013.12.002

[B10] Hampton S. Rabagliati H. Sorace A. Fletcher-Watson S. (2017). Autism and bilingualism: a qualitative interview study of parents' perspectives and experiences. J. Speech Lang. Hear. Res. 60, 435–446. 10.1044/2016_JSLHR-L-15-0348 28196376

[B11] Harris B. Barton E. E. Albert C. (2014). Evaluating autism diagnostic and screening tools for cultural and linguistic responsiveness. J. Autism Dev. Disord. 44, 1275–1287. 10.1007/s10803-013-1991-8 24186120

[B12] Hoff E. Ribot K. M. (2017). Language growth in English monolingual and Spanish-English bilingual children from 2.5 to 5 years. J. Pediatr. 190, 241–245. 10.1016/j.jpeds.2017.06.071 28803620 PMC5690817

[B13] Howard K. B. Katsos N. Gibson J. L. (2019). The school experiences of bilingual children on the autism spectrum: an interpretative phenomenological analysis. Res. Dev. Disabil. 87, 9–20. 10.1016/j.ridd.2019.01.008 30703680

[B14] Howard K. B. Katsos N. Gibson J. L. (2021). Practitioners' perspectives and experiences of supporting bilingual pupils on the autism spectrum in two linguistically different educational settings. Br. Educ. Res. J. 47, 427–449. 10.1002/berj.3662

[B15] Hudry K. Rumney L. Pitt N. Barbaro J. Vivanti G. (2018). Interaction behaviors of bilingual parents with their young children with autism spectrum disorder. J. Clin. Child Adolesc. Psychol. 47, S321–S328. 10.1080/15374416.2017.1286592 28323454

[B16] Humphrey N. Symes W. (2010). Perceptions of social support and experience of bullying among pupils with autistic spectrum disorders in mainstream secondary schools. Eur. J. Spec. Needs Educ. 25, 77–91. 10.1080/08856250903450855

[B17] Hussein A. M. Pellicano E. Crane L. (2019). Understanding and awareness of autism among Somali parents living in the United Kingdom. Autism 23, 1408–1418. 10.1177/1362361318813996 30486651

[B18] Iadarola S. Hetherington S. Clinton C. Dean M. Reisinger E. Huynh L.. (2015). Services for children with autism spectrum disorder in three, large urban school districts: perspectives of parents and educators. Autism Int. J. Res. Pract. 19, 694–703. 10.1177/1362361314548078 25192859 PMC4483151

[B19] Jegatheesan B. (2011). Multilingual development in children with autism: perspectives of south asian muslim immigrant parents on raising a child with a communicative disorder in multilingual contexts. Biling. Res. J. 34, 185–200. 10.1080/15235882.2011.597824

[B20] Jones D. R. DeBrabander K. M. Sasson N. J. (2021). Effects of autism acceptance training on explicit and implicit biases toward autism. Autism 25, 1246–1261. 10.1177/1362361320984896 33472382

[B21] Kandeh M. S. Kandeh M. K. Martin N. Krupa J. (2020). Autism in black, Asian and minority ethnic communities: a report on the first Autism Voice UK Symposium. Adv. Autism 6, 165–175. 10.1108/AIA-12-2018-0051

[B22] Kay-Raining Bird E. Genesee F. Verhoeven L. (2016). Bilingualism in children with developmental disorders: a narrative review. J. Commun. Disord. 63, 1–14. 10.1016/j.jcomdis.2016.07.003 27461977

[B23] Kay-Raining Bird E. Lamond E. Holden J. (2012). Survey of bilingualism in autism spectrum disorders. Int. J. Lang. Commun. Disord. 47, 52–64. 10.1111/j.1460-6984.2011.00071.x 22268901

[B24] Kim H. U. Roberti M. (2014). Tengo que habla español. Yo no entiendo ingles!”: a qualitative case study on a bilingual child with Autism Spectrum. J. Spec. Educ. Apprenticesh. 3:7.

[B25] Kinnear S. H. Link B. G. Ballan M. S. Fischbach R. L. (2016). Understanding the experience of stigma for parents of children with autism spectrum disorder and the role stigma plays in families' lives. J. Autism Dev. Disord. 46, 942–953. 10.1007/s10803-015-2637-9 26659549

[B26] Kremer-Sadlik T. (2005). To be or not to be bilingual: autistic children from multilingual families, in Proceedings of the 4th International Symposium on Bilingualism (Tempe, AZ),1225–1234.

[B27] Laasonen M. Smolander S. Lahti-Nuuttila P. Leminen M. Lajunen H. R. Heinonen K.. (2018). Understanding developmental language disorder-the Helsinki longitudinal SLI study (HelSLI): a study protocol. BMC Psychol. 6, 1–13. 10.1186/s40359-018-0222-7 29784061 PMC5963016

[B28] Leung C. (2010). English as an additional language: learning and participating in mainstream classrooms, in Conceptualising ‘Learning' in Applied Linguistics, eds Seedhouse P. Walsh S. Jenks C. (London: Palgrave Macmillan UK), 182–205.

[B29] Lumsden J. Ruchill Autism Unit. (2009). Modern languages and autism. Scottish Lang. Rev. 19, 13–20.

[B30] Mdlalo T. Joubert R. W. Flack P. S. (2019). The cat on a hot tin roof? critical considerations in multilingual language assessments. South Afr. J. Commun. Disord. 66, 1–7. 10.4102/sajcd.v66i1.610 31170786 PMC6556923

[B31] Montgomery L. Chondrogianni V. Fletcher-Watson S. Rabagliati H. Sorace A. Davis R. (2021). Measuring the impact of bilingualism on executive functioning via inhibitory control abilities in autistic children. J. Autism Dev. Disord. 10.31219/osf.io/x72ne. [Epub ahead of print]. 34406588 PMC9296418

[B32] Munroe K. Hammond L. Cole S. (2016). The experiences of African immigrant mothers living in the United Kingdom with a child diagnosed with an autism spectrum disorder: an interpretive phenomenological analysis. Disabil. Soc. 31, 798–819. 10.1080/09687599.2016.1200015

[B33] Nolte K. Fletcher-Watson S. Sorace A. Stanfield A. Digard B. G. (2021). Perspectives and experiences of autistic multilingual adults: a qualitative analysis. Autism Adulthood 10.1089/aut.2020.0067. [Epub ahead of print]. PMC8992916 36601639

[B34] Norbury C. F. Sparks A. (2013). Difference or disorder? cultural issues in understanding neurodevelopmental disorders. Dev. Psychol. 49:45. 10.1037/a0027446 22390668

[B35] Opitz B. Degner J. (2012). Emotionality in a second language: it's a matter of time. Neuropsychologia 50, 1961–1967. 10.1016/j.neuropsychologia.2012.04.021 22569217

[B36] Oxley E. De Cat C. (2019). A systematic review of language and literacy interventions in children and adolescents with English as an additional language (EAL). Lang. Learn. J. 49, 265–287. 10.1080/09571736.2019.1597146

[B37] Pacífico M. C. de Paula C. S. Namur V. S. Lowenthal R. Bosa C. A. Teixeira M. C. T. V. (2019). Preliminary evidence of the validity process of the Autism Diagnostic Observation Schedule (ADOS): translation, cross-cultural adaptation and semantic equivalence of the Brazilian Portuguese version. Trends Psychiatry Psychother. 41, 218–226. 10.1590/2237-6089-2018-0063 31644690

[B38] Papoudi D. Jørgensen C. R. Guldberg K. Meadan H. (2021). Perceptions, experiences, and needs of parents of culturally and linguistically diverse children with autism: a scoping review. Rev. J. Autism Dev. Disord. 8, 195–212. 10.1007/s40489-020-00210-1

[B39] Park S. (2014). Bilingualism and children with autism spectrum disorders: issues, research, and implications. NYS Tesol J. 1, 122–129.

[B40] Samadi S. A. McConkey R. (2015). Screening for autism in Iranian preschoolers: contrasting M-CHAT and a scale developed in Iran. J. Autism Dev. Disord. 45, 2908–2916. 10.1007/s10803-015-2454-1 25911978

[B41] Samadi S. A. McConkey R. Bunting B. (2014). Parental wellbeing of Iranian families with children who have developmental disabilities. Res. Dev. Disabil. 35, 1639–1647. 10.1016/j.ridd.2014.04.001 24814475

[B42] Selman L. E. Fox F. Aabe N. Turner K. Rai D. Redwood S. (2018). ‘You are labelled by your children's disability'–A community-based, participatory study of stigma among Somali parents of children with autism living in the United Kingdom. Ethn. Health 23, 781–796. 10.1080/13557858.2017.1294663 28277014

[B43] Shattuck P. T. Durkin M. Maenner M. Newschaffer C. Mandell D. S. Wiggins L.. (2009). Timing of identification among children with an autism spectrum disorder: findings from a population-based surveillance study. J. Am. Acad. Child Adolesc. Psychiatry 48, 474–483. 10.1097/CHI.0b013e31819b3848 19318992 PMC3188985

[B44] Szczepura A. (2005). Access to health care for ethnic minority populations. Postgrad. Med. J. 81, 141–147. 10.1136/pgmj.2004.026237 15749788 PMC1743229

[B45] Uljarević M. Katsos N. Hudry K. Gibson J. L. (2016). practitioner review: multilingualism and neurodevelopmental disorders–an overview of recent research and discussion of clinical implications. J. Child Psychol. Psychiatry 57, 1205–1217. 10.1111/jcpp.12596 27443172

[B46] UN General Assembly (2006). Convention on the Rights of Persons With Disabilities. https://www.google.com/search?sxsrf=AOaemvISHmRRJLiEIyK1wIPzuogyoM_gfg:1630218540370&q=New+York&stick=H4sIAAAAAAAAAOPgE-LQz9U3MC8rMFfiBLGM4o0szLS0spOt9POL0hPzMqsSSzLz81A4VhmpiSmFpYlFJalFxYtYOfxSyxUi84uyd7AyAgC5E7HrUQAAAA&sa=X&ved=2ahUKEwibwISozdXyAhUQ6XMBHepVDI4QmxMoATAqegQIPxAD New York, NY: UN General Assembly.

[B47] Valicenti-McDermott M. Tarshis N. Schouls M. Galdston M. Hottinger K. Seijo R.. (2013). Language differences between monolingual English and bilingual English-Spanish young children with autism spectrum disorders. J. Child Neurol. 28, 945–948. 10.1177/0883073812453204 22859698

[B48] Verhoeven L. (2007). Early bilingualism, language transfer, and phonological awareness. Appl. Psycholinguist. 28, 425–439. 10.1017/S014271640707023324175682

[B49] Wang J. Hedley D. Bury S. M. Barbaro J. (2020). A systematic review of screening tools for the detection of autism spectrum disorder in mainland China and surrounding regions. Autism 24, 285–296. 10.1177/1362361319871174 31431046

[B50] Wang M. Jegathesan T. Huber J. Minhas R. (2018). Raising children with autism spectrum disorders in monolingual vs bilingual homes: a scoping review. Early Lang. 39, 434–446. 10.1097/DBP.0000000000000574 29746381

[B51] Wire V. (2005). Autistic spectrum disorders and learning foreign languages. Support Learn. 20, 123–128. 10.1111/j.0268-2141.2005.00375.x

[B52] Yu B. (2013). Issues in bilingualism and heritage language maintenance: Perspectives of minority-language mothers of children with autism spectrum disorders. Am. J. Speech Lang. Pathol. 22:10. 10.1044/1058-0360(2012/10-0078) 23071196

[B53] Yu B. Hsia S. (2019). Inclusion of heritage language learners on the autism spectrum: lessons from second-generation parents. Int. J. Appl. Linguist. 29, 356–369. 10.1111/ijal.12233

[B54] Zhang J. Wheeler J. J. Richey D. (2006). Cultural validity in assessment instruments for children with autism from a chinese cultural perspective. Int. J. Spec. Educ. 21, 109–114.

